# Pollen-mediated gene flow and seed exchange in small-scale Zambian maize farming, implications for biosafety assessment

**DOI:** 10.1038/srep34483

**Published:** 2016-10-03

**Authors:** Thomas Bøhn, Denis W. Aheto, Felix S. Mwangala, Klara Fischer, Inger Louise Bones, Christopher Simoloka, Ireen Mbeule, Gunther Schmidt, Broder Breckling

**Affiliations:** 1 GenØk – Centre for Biosafety, Tromsø, Norway; 2 School of Biological Sciences, University of Cape Coast, Ghana; 3 National Institute for Scientific and Industrial Research, Zambia; 4 Swedish University of Agricultural Sciences, Sweden; 5 University of Vechta, Germany; 6 University of Bremen, Germany

## Abstract

Gene flow in agricultural crops is important for risk assessment of genetically modified (GM) crops, particularly in countries with a large informal agricultural sector of subsistence cultivation. We present a pollen flow model for maize (*Zea mays*), a major staple crop in Africa. We use spatial properties of fields (size, position) in three small-scale maize farming communities in Zambia and estimate rates of cross-fertilisation between fields sown with different maize varieties (e.g. conventional and transgene). As an additional factor contributing to gene flow, we present data on seed saving and sharing among farmers that live in the same communities. Our results show that: i) maize fields were small and located in immediate vicinity of neighboring fields; ii) a majority of farmers saved and shared seed; iii) modeled rates of pollen-mediated gene flow showed extensive mixing of germplasm between fields and farms and iv) as a result, segregation of GM and non-GM varieties is not likely to be an option in these systems. We conclude that the overall genetic composition of maize, in this and similar agricultural contexts, will be strongly influenced both by self-organised ecological factors (pollen flow), and by socially mediated intervention (seed recycling and sharing).

Maize (*Zea mays* L) is the cereal with the highest annual production worldwide with 1022 million tons produced on 183 million ha of land in 2014^1^. In its center of origin in Meso-America, but also in South America and in Sub-Saharan Africa, maize is a staple food crop and contributes significantly to calories and food security for millions of subsistence oriented small-scale farmers^2^,^3^.

Maize gene flow occurs through cultivator determined seed selection and mixing as well as through pollen transfer between individual plants and fields. Both traits are affected by the features inherent in the maize grown, agroecological circumstances and farmer’s practices. Gene flow occurs between all sexually compatible plants of maize types, i.e. in land races, commercial hybrids and eventual wild relatives^4^.

From its center of origin, farmers have for thousands of years experimented, spread, mixed and selected favorable maize plants for different environmental conditions, which provide the crop biodiversity that currently exists^5^,^6^,^7^. This diversification process is based on a long history of informal systems for seed exchange. High diversity in maize is found on the community level rather on individual farms^8^, and this diversity may also to some extent be an unintended result of farmers’ practices^9^. In fact, at the larger regional scale, maize crop biodiversity results as an emergent property of individual farmers’ management in combination with the surrounding ecological conditions, abiotic as well as biotic factors.

Few countries have adopted genetically modified (GM) crops for commercial use on the African continent. South Africa, Sudan and Burkina Faso are currently the only African countries where GM crops are grown commercially^10^. Egypt grew insect resistant Bt-maize from 2008 but stopped this in 2011^10^. Kenya, Nigeria, Uganda, Cameroon, Egypt, Ghana, Malawi and Uganda perform field trials to evaluate GM crops for their agricultural contexts^11^,^12^,^13^. With a potential increase in the adoption of GM crops in Africa in the coming years, better understanding of the implications of introducing GM crops into small-scale farming systems is needed. An increasing share of countries are adopting labelling requirements for food with GMO content, to ensure consumer choice. Thus, the potential for exporting maize to markets with premium prices (e.g. organic), requires control over rates of cross-hybridisation between GM and non-GM maize.

Countries that require labeling of food containing GMO are using different thresholds. In South Africa, the Consumer Protection Act requires that foodstuff containing at least 5% GMO must be labelled (the Consumer Protection Act 2008, April 2011). In the EU, food products containing more than 0.9% GMO must be labelled. Within the EU there is zero tolerance to GM in products certified as organic, but in the US 5.0% GM content is allowed in products labelled as organic^14^. The Cartagena Protocol on Biosafety (http://bch.cbd.int/protocol/), signed by 170 countries, regulates transboundary movement of GMOs (LMOs in the Protocol). The Protocol has implemented the mandatory requirement to produce information such as labeling of GMOs that crosses national borders, and to ensure biosafety capacity building in all parties^15^.

Large-scale agriculture has been the basis for developing segregation measures and co-existence policies in many countries^16^. The use of GM crops in small-scale agriculture systems however brings with it new questions, e.g. with regard to co-existence between farmers who plant GM and non-GM crops^16^,^17^,^18^. Understanding and control over gene flow are central to these issues.

Progress has been made in understanding gene flow in industrial agricultural settings^19^,^20^,^21^, but by contrast our knowledge of gene flow is very restricted for cases where small-scale and subsistence agriculture dominates the agro-ecosystems. A larger part of empirical gene flow investigations and models focuses more on local, case specific factors than on relations involving regional field-to field gene flow (for a review, see ref. 22, for a generic model approach with relatively high parametrisation requirements see ref. 23). Specifically adapted to maize is the MAPOD approach, which also requires a high level of parametrisation and aims at predicting pollen flow in single fields or within a smaller number of fields in a specified location^24^,^25^. Acquired knowledge from industrial-scale production may have limited applicability for considerably more heterogeneous agricultural conditions, especially if the farmers have significantly diverging agricultural practices and understanding (e.g. whether they see cross-hybridisation as a problem or not, and how they acquire and use seed).

Gene flow studies in maize have emphasized the role of pollen movement and cross-pollination^26^,^27^. The degree of cross-pollination in maize depends on the spatial distance between different maize fields, and timing and local conditions during flowering. Most of the cross-pollination in maize is found within the first 30 m^28^, but includes a long tail with low cross-pollination occurring over several hundred meters^20^ (see also studies referenced in Fig. 1). Cross-pollination is still not zero at 650 m^29^ and may also occur up to 800 m distance when climatic conditions are favorable for pollen flow^30^. Because of their greater degree of genetic heterogeneity, open pollinated varieties (OPVs) (including so called landraces and certified OPVs, both of which are more commonly used by smallholders than by large-scale farmers) flower over a longer period than hybrid varieties^31^ and have higher outcrossing rates than hybrid cultivars^32^.

Modeling and meta-analysis have highlighted the following key variables for quantification of gene flow by pollen: (i) the distance between fields, (ii) the size/width of the recipient field, (iii) the synchronism of flowering, and (iv) the width of buffer zone planted around the recipient field^33^,^34^.

Quantitative recommendations to avoid pollen flow in maize cultivation may be of limited or no use when (i) fields are so small so that isolation barriers would consume a significant part of individual fields, and (ii) farmers practice seed sharing to a significant extent.

In commercially oriented maize farming, hybrid seed is commonly bought new every year and the farmer relies on the seed industry for providing them with suitable maize varieties (this is often referred to as the formal seed system). In contrast, subsistence oriented smallholders all over the world rely to a considerable extent on distribution and use of maize in so called “informal seed systems”, where farmers themselves develop, re-use and share seed. Farmer to farmer seed exchange represents renewal of local varieties/land races and serves as an inexpensive and reliable source of seed in communities where a formal seed system does not exist or cannot be trusted to deliver seed of desired quality^6^,^7^,^31^,^35^,^36^,^37^. Seed sharing, however, also makes people effective vectors of genetic material; over large distances, eventually across natural and regulatory borders. Modeling has shown that seed flow may lead to a much wider diffusion of genes than expected by pollen movement alone^38^. Seed sharing also effectively links the gene pools of individual maize fields into communal or regional metapopulations^36^,^39^. How transgenes, if introduced, would diffuse, evolve and ultimately impact such a system is largely unknown.

In this study we present detailed maps generated from GPS data on sizes and spatial arrangement of maize fields in three Zambian communities. Key features of the fields (position, size, and distances to other fields) were used to parameterize a mathematical model that estimates pollen flow between fields. We simulate the outcomes of different scenarios of pollen flow in small-scale agriculture (for a Zambian context, fields smaller than 5 ha) under the presumption that farmers were adopting GM maize (Zambia has not adopted GM crops at present). Thus, we gauge the probability for potential unintended transgene introgression amongst neighboring fields in a small-scale maize farming context, with seed exchange as a further complicating factor. We vary the adoption rate of GM and the degree of synchrony in flowering between fields, and apply these scenarios to GPS and interview data from the three Zambian communities of Chongwe, Chipapa and Mumbwa. The results from the questionnaires and the outcome of the modeling of pollen-flow are analyzed jointly, and we discuss the interactions between pollen and seed flow for the potential spread and mixing of maize genes in a smallholder farming context.

## Materials and Methods

### Ethics statement

Prior to the study, for the approval of the field work, we approached the District Agricultural Coordinating Officers (DACO) who approved our methods and suggested suitable areas to carry out the field work. They further linked us with Camp Officers (CO) who accompanied us in the field and helped us in identifying farmers to interview and their fields. The CO work with these farmers on a regular basis and are familiar with the local context. Before each interview or group discussion, the CO explained the research context to the farmers and asked for permission that we could a) interview them, and, b) measure their fields. All individual farmers gave us their informed consent. Each farmer was also informed that she/he did not have to answer any questions if she/he did not want to, and that it was possible to withdraw from the study at any time. The farmers were furthermore informed that all their statements would be presented to third parties anonymously. Also, for the sake of ensuring personal integrity, individual farmers were not connected with fields mapped. As some farmers were illiterate, and many felt uncomfortable with writing, the farmers gave their oral consent to participating in interviews. The questions and set-up for the interviews were based on an already established methodology for qualitative and participatory research, used and published in a similar agricultural context in South Africa^39^. When the information collected in questionnaires is exclusively anonymous, there is no obligation to have the project notified under the Norwegian Data Protection Official: If information is registered anonymously, the project is not subject to notification (http://www.nsd.uib.no/personvern/en/notification_duty/). Thus, the methods were carried out in accordance with the approved guidelines.

### Mapping of fields

We mapped the spatial patterns of cultivated fields in the maize farming communities of Chongwe, Chipapa and Mumbwa in Zambia, using handheld GPS devices (Garmin 62S) in March and April 2012. This type of direct field mapping is precise, but time-consuming and thus limited. For mapping larger regions, remote sensing data and other suitable proxy-data may be more suitable, if available^40^. For the relevant regions of Zambia this is not the case.

We walked in a grid of transects to make sure that all fields were detected within the predetermined area and made ‘detours’ by following each maize field margin with GPS waypoints to describe the exact shape and size of each individual field. The coordinates (projection UTM, GCSArc1950) of the waypoints of each field were transformed to a polygon layer by means of ArcGIS 10.0, ESRI (Environmental Systems Research Institute, 2011): ArcGIS Desktop: Release 10. Redlands, CA. Or URL: (http://www.esri.com/software/arcgis/arcgis10), and field centroids and acreage were calculated. Analysis of the spatial distribution, and average distances to nearest field neighbors with respect to field centroids were performed using our own (B. Breckling) computer programmes written in SIMULA^40^,^41^.

### General description of the model

**Figure 1:**
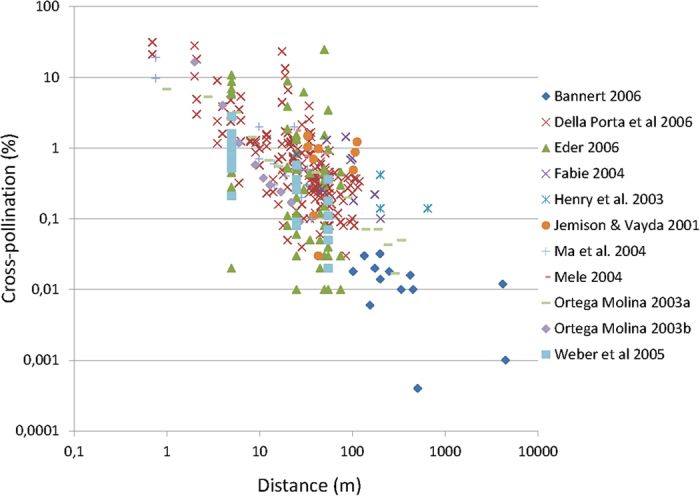
Measured values of gene flow. Given on a log-log scale, as obtained by 11 studies documented in the peer reviewed literature.

We used a model that calculates gene flow within a single season between spatially distinct populations of maize on a landscape level, i.e. on a community or regional scale^42^,^43^,^44^. The model simulates cross-pollination and uses each field both as a source and as a receptor for cross-fertilisation within a field-to-field distance for which gene flow was confirmed in peer reviewed studies (summarized in Fig. 1) – and for the time of overlapping flowering. The outcomes of the model are statistically robust trends of gene flow between fields. The core of the model is a dispersal kernel used to calculate the functional relation of field distances, average cross-pollination decreasing with distance, and random characteristics of the process. For this purpose, we chose parameters in accordance with the best documented and comparable empirical studies of gene flow between maize fields in the peer-reviewed literature^45^,^46^,^47^,^48^,^49^,^50^,^51^,^52^,^53^,^54^,^55^,^56^. We compiled the study data in a graph showing amount of gene flow over distance on a double logarithmic scale (Fig. 1). A coherent decrease of gene flow with increasing field-to-field distance is apparent. The use of a log-linear distance relationship and a log-normal distribution of the random characteristics is also in accordance with pollen dispersal models^57^. To derive a dispersal kernel for gene flow we used the log linear regression as a mean and adapted a log normal distribution as a goal function. The simulated duration of flowering was set to 10 days. In order to include the random onset of flowering, we calculated three different scenarios using a normal distribution with a standard deviation of: (i) 18 days (largely asynchronous flowering), (ii) six days (intermediate) and (iii) one day (largely synchronous flowering). The random onset of flowering of each single field made it necessary to calculate the gene flow only for the days of overlapping flowering. The parameterisation of gene flow per day was adjusted to meet the dispersal characteristics as measured for the entire flowering period (Fig. 1). The field-to-field gene flow for all distances was combined from the summed up daily values resulting from the days of overlapping flowering periods.

### Key parameter and model calculations

The model uses the following variables/parameters: (i) field size, (ii) field centroid distances to neighboring fields, (iii) normally distributed difference in the onset of flowering period between maize fields, and (iv) number and size of GM maize fields within a population of non-GM maize fields. Field sizes were incorporated into the calculations by using a size-proportional weighting factor. A doubling of the source field size doubled the gene flow rate; doubling of the sink field size reduced the gene flow rate by 50%. This abstraction was derived from the consideration that overall production of released pollen is proportional to the number of plants that is proportional to the field area, i.e. grossly homogeneous across the field. The model targets to calculate regional conditions rather than isolated fields, and can cope with more than 100.000 fields for a model run. To achieve this, the model abstracts from details of the field geometry by using field centroids to specify mean distances and the field size to set donor and receptor potential in proportion. The use of centroid distances is shown to provide more reliable estimates for the average rate of pollen flow than the use of edge-to-edge distances^42^. A recent compilation of pollen dispersal data^45^ confirms the functional relationship and its random component as we used it for the developed dispersal kernel. Key calculations of the model are

(1) Aggregation of field-to field gene flow for the season from daily values


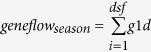


(2) One day calculation


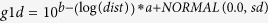


(3) Elimination of close-distance extreme values.

At small distances, the random function may generate too large values, which do not occur in empirical datasets. To avoid this, these values are reduced by the function


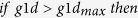



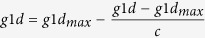


where ***dsf*** = number of days of synchronous flowering (0–10); ***g1d*** = gene flow (%) occurring at one day; **log(*****dist***) = base 10 logarithm of the field centroid distance; ***b*** = base 10 logarithm of the mean gene flow at a distance of 1 m (value: 1.1); ***a*** = slope of the log-linear decrease of gene flow with distance (value: 1.2); **NORMAL** = normal distribution with a median of 0.0 and a standard deviation of sd.; ***sd*** = standard deviation of a normal distribution (value: 1.2); ***g1d***_***max***_ = maximum gene flow to occur at 1 day. We used the value 1.2 (~15.8%); ***c*** = reduction factor (value: 18.0).

The model calculates gene flow at distances between 1 m and 4500 m, i.e. well above the relevant distances in our dataset. The used parameterisation implies that for an increase of the field centroid distance of one order of magnitude, the gene flow decreases 1.2 orders of magnitude on average. Outcrossing of genes and introgression can be considered as synonymous as long as there is no specific incompatibility or other forms of selection involved for the relevant gene of interest. Positive or negative fitness (selection) is not included in the model at present.

### Modelled scenarios of GM introduction

We used different scenarios to simulate gene flow for each of the three communities of Chongwe, Chipapa and Mumbwa and calculated rates of pollen flow after the introduction of GM maize plants in (i) the single largest field (2–9 ha), (ii) 10% of the fields, and (iii) 40% of the fields. For the latter two scenarios, fields containing GM maize were randomly selected. For each of the three scenarios, we varied the synchrony of a ten day pollen shedding period (onset of flowering of each field), from full synchrony with one day standard deviation, six days standard deviation and a standard deviation of 18 days. The simulations were repeated ten times with different random start values to assure statistically independent model runs with different realisations of random impact. The results of the ten model runs were then averaged. The 3 × 3 × 3 = 27 chosen scenarios modeled (i.e. the scale of GM maize introduction x flowering synchrony x the three community sites) cover a range of alternatives and inform about the variability that is involved in the pollen flow processes. For all scenarios, it has to be kept in mind that the percentage of modified alleles is counted. It is assumed that the focal gene is available in one copy in the hybrid varieties. Therefore, the sown GM varieties are indicated to have 50% GM alleles as hybrid breeding aims are that all individuals have one. For gene flow across more than one generation, there would be the implication that a number of individuals can occur with two copies of the focal gene.

### Farmer and seed management survey

To gather information on management practices, we performed structured interviews with farmers (n = 15–35 per community); asking how many fields they had, what maize varieties and other crops they grew, and how they acquired, selected and stored their maize seed. Further, if and how many of them recycled (i.e. re-sowed harvested) maize seed, if and with whom they shared maize seed, and how far away farmers they shared seed with grew their maize. Detailed responses were recorded in questionnaires. We focused the farmer interviews on the maize fields that we mapped with GPS. However, many of the farmers also had gardens where they planted partly overlapping and partly different maize varieties. Seed used in gardens and seed used in the field would in practice be mixed for most farmers, but we do not address this complexity in the present work.

## Results

### Spatial field mapping

**Figure 2:**
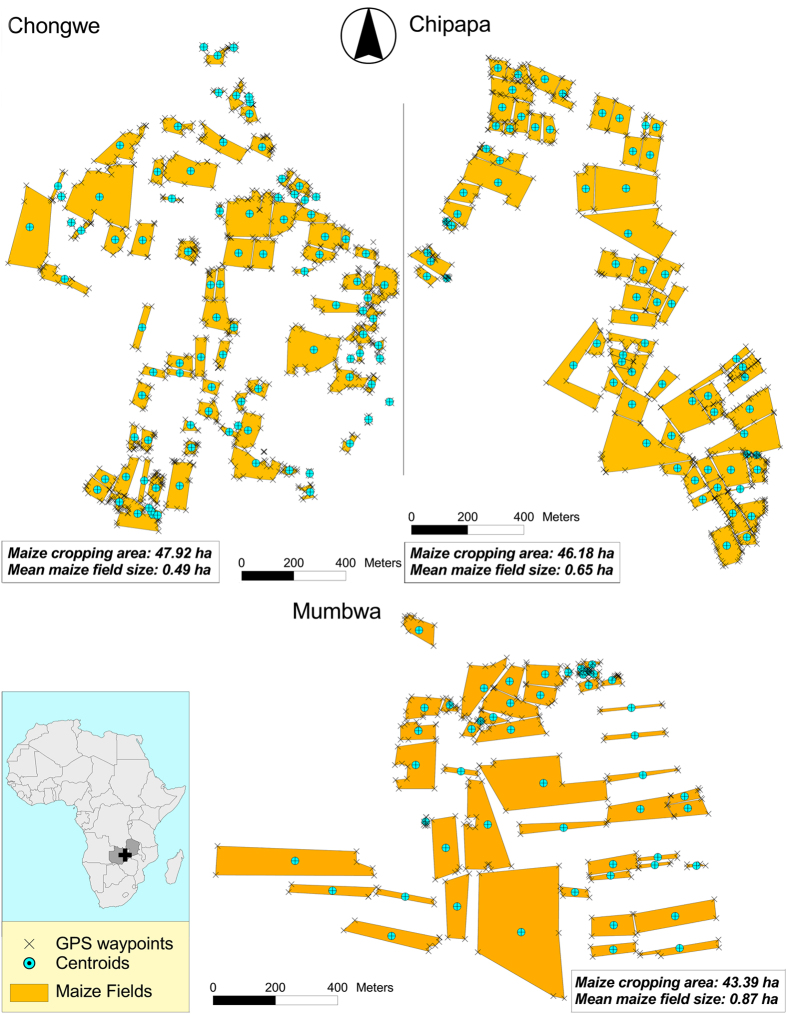
Maps derived from GPS coordinates of maize fields. Fields are shown in orange, in communities Chongwe (top left), Chipapa (top right) and Mumbwa (bottom). All GPS waypoints and centroids for each field are indicated. The software used for generating the maps: ArcGIS 10.0 by ESRI), URL: http://www.esri.com/software/arcgis/arcgis-for-desktop.

**Figure 3:**
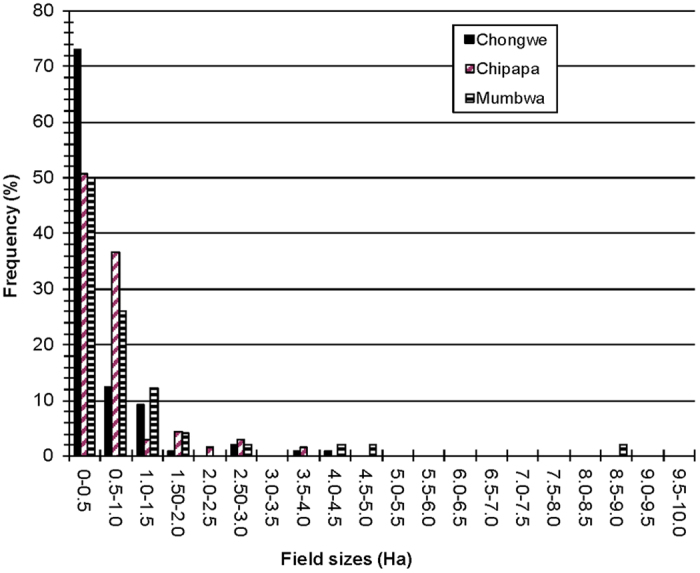
Sizes of fields in communities Chongwe, Chipapa, and Mumbwa.

**Figure 4:**
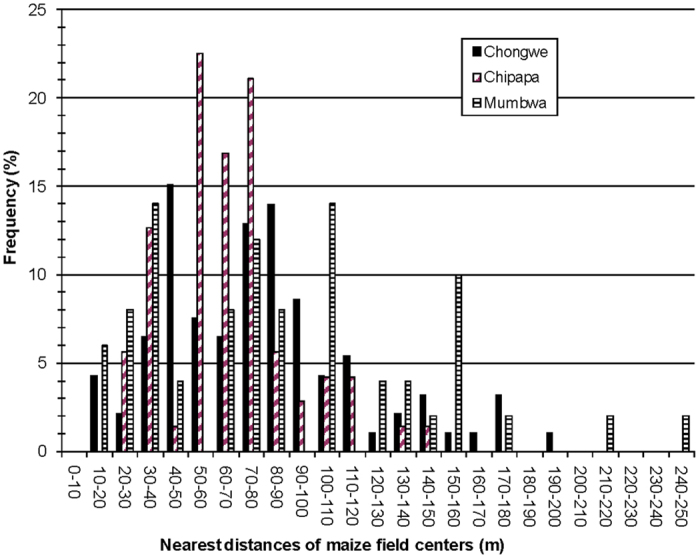
Nearest neighbor distances of maize fields in communities Chongwe, Chipapa, and Mumbwa. The distances are calculated between the field centroids.

In the Zambian communities of Chongwe, Chipapa and Mumbwa, 97, 71 and 50 maize fields were found, each within an area of 1 km^2^, respectively, showing a high cultivation density of fields, implying also close proximity between them. In Chongwe, the mapped maize fields had an average size of 0.49 ha and occupied 48% of the total area mapped, while in Chipapa and Mumbwa, the mapped maize fields had an average size of 0.65 ha and 0.87 ha respectively. In Chipapa and Mumbwa, maize fields occupied 46% and 43%, respectively, of the total area mapped (Fig. 2). For all the three communities, more than 50% of the fields were smaller than 0.5 ha and few fields were larger than 2.0 ha (Fig. 3). With few exceptions, mostly found in Mumbwa, all maize fields in the studied communities had neighboring fields that were within 10 m away and fields were often separated by narrow tracks only (1–5 m). Field centroids typically had distances less than 100 m to the nearest neighboring field centroid (Fig. 4).

### Pollen flow simulations within a single season

The rate and variation of pollen flow between fields were highly similar between the three communities, indicating that community arrangements and gene flow rates were comparable in Chongwe, Chipapa and Mumbwa.

### Scenario I – pollen flow from a single large farm growing GM maize

**Figure 5:**
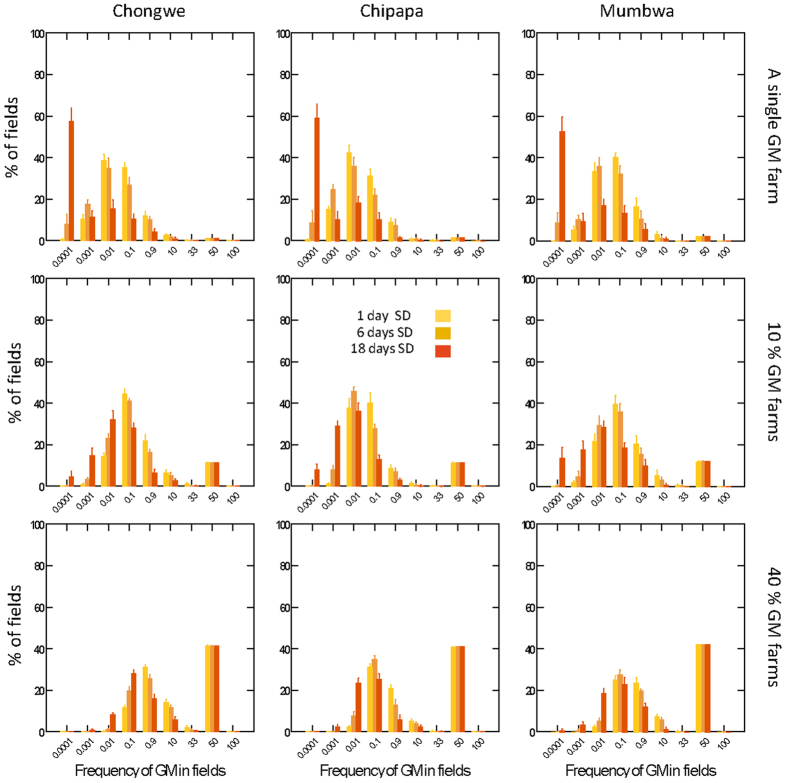
Model output: Frequency distribution of GM material in maize fields after one season of cross-pollination. Source of GM material is from a single large GM source field (top), from 10% of the fields (middle), and from 40% of fields (bottom), with full synchrony 1 day standard deviation (SD) in the onset of flowering (left column, yellow), or a SD of 6 days (middle column, brown) or 18 days SD (right column, red). Error bars show 95% confidence intervals. The calculations were based on 10 model repetitions with different random start values, based on the actual field distribution as mapped with GPS in the communities of Chongwe, Chipapa and Mumbwa.

If a single farmer with the largest field planted GM maize on this field, most fields across the community would contain GM pollen at a level around 0.01 to 0.1% (Fig. 5). Due to cross-pollination, a few fields with conventional (i.e. non-GM) maize in Chongwe and Mumbwa would contain GM at a level between 0.9 and 10% (Fig. 5 upper panels), when the onset of flowering was well synchronized.

### Scenario II – pollen flow when 10% of farmers grow GM maize

If 10% of the farmers (randomly scattered around the community), grew GM, most of the conventional fields would contain GM at a level of 0.001–0.9%. In Chongwe and Mumbwa, about 5% of the fields would contain between 0.9 and 10% GM (Fig. 5, middle left and middle right panels, respectively). The simulations for Chipapa showed slightly lower cross-hybridisation than the other two communities. Note that in Fig. 5, middle panels, about 10% of the fields contain more than 50% GM. These are the fields sown with GM plants, but with a small fraction of incoming non-GM pollen, thus reducing the amount of GM to slightly below 100%.

### Scenario III – pollen flow when 40% of farmers grow GM maize

If 40% of the farmers (randomly scattered around the community) grew GM, the proportion of conventional fields that contained between 0.9 and 10% GM material would have increased to about 15, 5 and 7% of the fields for Chongwe, Chipapa and Mumbwa, respectively, at full synchrony (Fig. 5 lower panels). With lower degree of synchrony, e.g. with a flowering period offset of 18 days (Fig. 5), cross-pollination was still significant between fields (Fig. 5 lower panels, red bars). Note that in Fig. 5, lower panels, about 40% of the fields contain more than 50% GM. These are the fields sown with GM plants, but with a small fraction of incoming non-GM pollen, thus reducing the amount of GM to slightly below 100%.

### Seed saving and sharing

**Figure 6:**
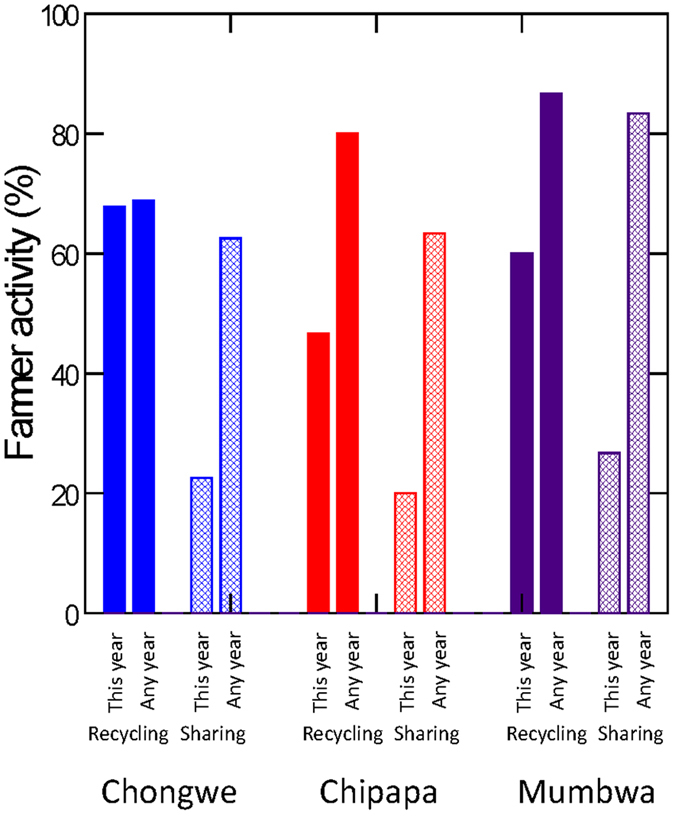
Recycling and sharing of seeds. Proportion of farmers that recycled seeds (this year or any year) and shared seeds with other farmers (this year or any year) in communities Chongwe, Chipapa and Mumbwa, respectively.

**Figure 7:**
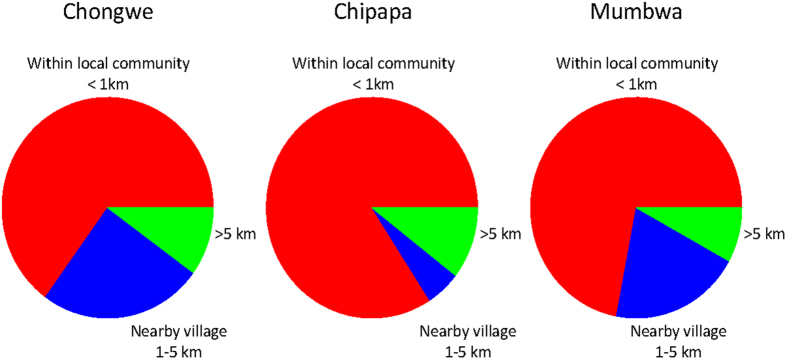
Travelling distance of shared seeds in Chongwe, Chipapa and Mumbwa.

The majority of farmers reported that they planted commercial hybrid maize, but locally developed open pollinated varieties (OPVs) were also in use. Not only OPVs but also hybrid seed were commonly recycled, indicating that reported levels of ‘hybrid seed’ planted includes a substantial amount of seed material taken from previously planted hybrids. Questionnaire responses from the three communities showed that 48–67% of the farmers from the three Zambian communities recycled seed between 2011 and 2012, and 69–87% said they normally did so (i.e. any year) (Fig. 6). For the 2011–2012 season, 20–27% of the farmers reported that they had shared seed with other farmers, and 63–83% of farmers reported that they normally shared seed (any year). Most of the sharing was reported to occur within the local community within a range of 800 m away (Fig. 7). But 25, 5.3 and 20% of the farmers had also shared seed with nearby villages (1–5 km away); and 10, 10.5 and 8% had shared seed with even more distant communities (5–100 km away), in Chongwe, Chipapa and Mumbwa, respectively (Fig. 7).

## Discussion

The study presented here assesses different factors influencing gene flow in maize cultivation. We exemplify African conditions by the investigation of three villages in rural Zambia where small-scale subsistence cultivation prevails. The cultivation conditions differ considerably from those for which biosafety investigations of genetically modified maize were done in North America and Europe. In Zambia, as well as in other countries where maize is cultivated for subsistence, the social conditions include the widespread practice of recycling and sharing of seed. This largely increases the potential level for human mediated gene flow, which may add to and interact with pollen mediated gene flow.

The questionnaire responses from the Zambian farmers confirmed that seed sharing and exchange of maize was a common phenomenon. Thus, extensive human mediated seed flow could be expected. In addition, most of the maize fields that were mapped occurred in immediate proximity to one or several neighboring fields, often less than five meters apart, and rarely with any physical barrier like a bush, tree or fence between them.

Given the physical arrangements of fields, and the seed management pattern that we report; the potential for significant cross-hybridisation and gene flow between used maize varieties is high.

The mathematical model we used was designed to enable *regional* estimates of pollen mediated rates of gene flow, rather than predictions for single fields, which has been the focus of previous developments^21^,^23^,^24^. These have a greater potential to explain local variation. The approach we used^40^,^58^,^59^ reduces parameter requirements, which would not be available for larger regional contexts. However, we can elaborate expectations for spatially larger contexts^60^. The dispersal kernel used in our model provides a homogeneous functional approach to cover field centroid distances from 1 m to 4500 m, including a summarized variability of the pollen flow.

The modelling approach uses existing knowledge for the parametrisation of a dispersal kernel which was applied to the local geographic conditions of the three Zambian villages. There is a high variability involved in wind pollination, making it difficult to predict cross-pollination for single fields. However, from empirical data the extent of variation can be estimated, and the larger the number of fields involved, the more the process can be assumed to be levelled out according to the law of large numbers.

The results from our model, where we calculate how transgenic pollen would cross-hybridize into non-GM fields under a range of different scenarios, show that, during a single season, levels of GM material in fields not intentionally planted with GM, can be expected in the range of 0.01 and 1%, but may reach about 10% at the highest. Even a single large field (in this context 2–9 ha) planted with GM maize for a single season would cross-hybridize into non-GM at a level up to about 1%. If the cultivation pattern is repeated, pollen-mediated gene flow would accumulate and reach higher levels within a few years.

The model also shows that the initial fraction of GM maize grown, and the degree of synchrony in flowering between GM and non-GM maize had expected effects on the rate of pollen flow: larger fractions of GM fields and better match in flowering synchrony lead to a larger number of fields with higher percentages of GM material in non-GM harvests (summarized in Fig. 5).

The modelling study allows us to point out effects relevant for a multi-year context. For example, if the harvest of a small field that received a larger extent of gene flow is used in subsequent year to sow a larger field elsewhere, rapid changes in the increase of frequency rates in genetic composition may result.

Our results indicate that the recommended isolation distances between fields proposed in Europe to avoid gene flow, typically 200 m (15–800 m)^19^, would not be easily implemented without significantly changing agricultural practices and landscapes in regions similar to the studied Zambian communities. Even the centroid distances between fields (center-to-center distance) were smaller in the present study than the suggested distances to avoid pollen from cross-hybridizing between fields in many European countries. In Chongwe, Chipapa and Mumbwa, 89%, 77% and 60% of field centroids (that clearly will be more distant from each other than the field edges) occurred within a 100 m range of the nearest other field centroid, respectively.

To determine the wider applicability of the findings presented here it is now crucial to gather more data on field sizes and arrangements in other regions. Ghana and South Africa have similar features in their small-scale farming systems, e.g. less than 10 m separation of maize fields and recycling of seeds^40^,^61^,^62^.

For modelling of long-term effects, the selection coefficient (fitness) of the incoming gene/trait/plant will be crucial. Even a trait that gives the plant a slightly improved fitness may have strong impact on the long-term diffusion of the gene that controls that trait^38^. For example, positive natural selection of a GM plant with a trait like Bt toxin is likely where target insects are present^63^, or if a drought tolerant plant (GM or non-GM) is challenged with water stress. GM maize plants are modified to be agronomically superior in their context, either with protection to insect pests (Bt-plants), with tolerance to agrochemicals (HT-plants) or drought conditions. These three traits are already stacked into a single maize hybrid prepared for the African continent (South Africa). Such hybrids might have a positive fitness and may thus spread and expand in agricultural systems that reuse their seeds.

The vulnerability of a field for gene introgression due to pollen flow is inversely proportional to the size relation of the receiving field to the surrounding donor fields^33^,^34^. Small fields have less ‘volume’ to dilute the fraction of incoming pollen. Some authors have claimed that fields larger than 5 ha do not need any isolation distance^22^. In the studied Zambian communities, only one field out of the 218 mapped was larger than 5 ha, and more than 75% of the fields were smaller than 1 ha. This situation is similar to most communities in Zambia in terms of size and isolation distances of fields for small-scale farmers (smaller than 5 ha). FAO data on farm sizes in Sub-Saharan African countries indicates that this situation applies widely across Africa. For example, the average farm size in Uganda is 1.12 ha, Tanzania 1.5 ha and Kenya 0.86 ha, respectively^64^.

The timing in flowering between the relevant maize populations will determine the potential pollen cross-hybridisation. If the environment and cultural practices permit, this can be used as a measure to avoid pollen flow^65^. In the studied Zambian communities, where farmers plant maize under rain-fed conditions, maize is typically planted after the first rain following a longer period of cool dry and hot dry seasons from May to July and August to October, respectively. Therefore, many farmers plant more or less at the same time when the rainy season starts in November. Thus, maize cultivation is regionally largely synchronized. With the limited possibility of adapting planting dates under the practice of rain-fed agriculture, planting varieties with different time to flowering might be an alternative way to minimize cross-pollination, although the possibilities to implement and control this in practice would need to be further studied. The option might however not be sustainable since the differences between the varieties would be reduced by cross-hybridisation and exchange of seed among farmers.

The use of open pollinated varieties, (both locally developed – termed ‘gankata’ in Zambia – and purchased OPVs), which have higher outcrossing rates than hybrid cultivars^32^ may increase cross-hybridisation by pollen among fields and farms.

The vital role of seeds as an additional vehicle for gene flow must not be underestimated^26^. In particular, the human-driven gene flow through intentional and unintentional seed movement is relevant. The results presented here show that more than 60% of the farmers did save and share their seed. Other publications confirm the importance of sharing and re-using seed in other resource-constrained smallholder communities in particular^35^,^62^,^66^,^67^,^68^. These practices serve important roles for farmers, including a continuous and reliable seed supply in smallholder communities over the world^37^,^69^, and also to maintain and develop crop diversity in response to pests, climatic conditions and farmer’s preferences^6^,^67^,^70^.

The practice of re-using seed was an important part of the local food security and independence, according to the interviewed farmers. Both local maize varieties and commercial (hybrid) maize varieties were re-used. This latter practice constitutes a link between the informal and the formal seed system, as also documented elsewhere^16^,^71^,^72^,^73^.

In South Africa, GM maize is grown both in large and small scale, and seeds from formal and informal sectors are frequently mixed in smallholder systems. Thus, specific hypotheses on cross-hybridisation and introgression of transgenes could be tested there. A study from the province of Eastern Cape, South Africa, showed that transgenes remained in farmers’ fields 6 to 11 years after they were purposely planted, and continued to spread at low levels without control^16^. Transgenic seeds were also kept for recycling by smallholder farmers^62^. This illustrates how transgenes may enter into subsistence maize locally adapted genepools and stay there over time.

To estimate outcrossing rates require an understanding of the mechanisms that influence gene flow quantitatively. This can be done with modelling and is of interest scientifically as well as for policy and regulation. For example, in case a transgenic maize plant expressing Bt-toxin is hybridizing with a non-Bt plant, and recycled, the outcome would likely be a plant that expresses Bt-toxin at a low level. That would represent increased risk for unwanted resistance development in target insects, and also oppose the high-dose/refugia strategy.

Notably, once a transgenic gene or trait is out, it is almost impossible to eliminate it from the regional seed pool. The task of removing transgenes from a subsistence farming system (like the one in Eastern Cape) would be highly difficult, as it would require control of innumerable informal seed storages^62^. A clean-up process might also lead to a loss of locally selected genes and traits.

Given that smallholders often recycle and share seed, genes will not only diffuse locally but also regionally since sharing of seed also happens over longer distances (compared to the range of pollen flow). One illustrative case in the community of Chongwe showed that sharing of seeds within a family, from mother to daughter, moved seeds over a distance of about 100 kilometers, after the daughter moved to another village. Seed sharing happened relatively often also between communities/villages: 16–35% of the farmers in the three villages had shared seeds with farmers outside their own community, i.e. a distance of 1–100 kilometers. A recent modeling study showed that seed flow between farmers contributes to a much wider diffusion of genes (transgenes in Mexico) than expected from pollen flow only^38^.

The practices of re-use and sharing of hybrid as well as OPV seed documented in the present case is not limited to the studied communities, or to Zambia alone, but widespread across Africa^16^,^74^.

Most African countries, including Zambia, have not adopted GM maize. Therefore, apart from a scientific contribution to the understanding of gene-flow in small-scale farming in general, this study also contributes relevant information at a Zambian or wider African policy-level: the physical arrangements and sizes of fields, as well as farmer seed saving and sharing, will significantly affect the rate of gene flow.

Ecological factors controlling gene flow rates and recycling/sharing practices for seed can be considered as country specific relevant topics for risk assessment. Country specific evaluations should take into account the efficiency of containment measures, pest resistance management applicability, as well as transboundary movement regulation compliance in a crop variety admission context.

In conclusion, we show through modelling that smallholder Zambian farmers work in a system where high rates of pollen flow leads to extensive cross-hybridisation of maize varieties used in the field. In addition, farmers recycle and share maize seeds. This increases gene flow locally, but also increase the distance that genes travel. More data is needed to evaluate how representative the studied communities are for other small-scale farming areas in Zambia and other African countries. Future studies should address the combinatory effect of ecological (e.g. pollen flow) and social (e.g. seed management) processes, quantitatively. Seed saving and sharing needs to be co-analyzed and integrated into models of gene flow to understand what happens at different scales: from local to regional, national and transnational levels, over time.

## Additional Information

**How to cite this article**: Bøhn, T. *et al*. Pollen-mediated gene flow and seed exchange in small-scale Zambian maize farming, implications for biosafety assessment. *Sci. Rep*. **6**, 34483; doi: 10.1038/srep34483 (2016).
